# Characterization of the Disinfectant Resistance Genes *qacEΔ1* and *cepA* in Carbapenem-Resistant *Klebsiella pneumoniae* Isolates

**DOI:** 10.4269/ajtmh.23-0247

**Published:** 2023-12-11

**Authors:** Xiaoli Liu, Lin Gong, Ernan Liu, Changfeng Li, Yimei Wang, Jiansheng Liang

**Affiliations:** ^1^ Department of Disinfection and Pest Control, Wuhan Center for Disease Control and Prevention, Wuhan, China;; ^2^ Discipline Inspection Division, Wuhan Center for Disease Control and Prevention, Wuhan, China

## Abstract

The emergence and wide global spread of carbapenem-resistant *Klebsiella pneumoniae* (CRKP) isolates are of great concern. This multicenter study aimed to investigate the molecular characteristics of CRKP isolates from inpatients in Wuhan, China. From June 2018 to March 2019, 74 nonduplicated CRKP clinical isolates were collected from six hospitals in Wuhan. We determined the minimum inhibitory concentrations of 18 antibiotics and used real-time polymerase chain reaction to detect the presence of disinfectant resistance genes *qacEΔ1* and *cepA*. Pulsed-field gel electrophoresis was conducted to assess the genetic relatedness of isolates. Among the 74 CRKP isolates, the rates of resistance to carbapenems were high: 93.2% to ertapenem, 90.5% to imipenem, and 87.8% to meropenem. All isolates were resistant to at least one carbapenem antibiotic. Of the 74 isolates, 64.9% (48/74) were positive for *qacEΔ1* and 93.2% (69/74) for *cepA*. *QacEΔ1* and *cepA* were detected concomitantly in 46 isolates (62.2%), whereas only 4.1% (3/74) had no disinfectant resistance genes. Pulsed-field gel electrophoresis analysis clustered the 46 CRKP strains co-producing *qacEΔ1* and *cepA* into 15 different clonal clusters (Types A to O). The most common clonal clusters were Type C (41.3%), Type E (13.0%), and Type J (8.7%). The study showed high rates of resistance to most antibiotics and high frequency of *qacEΔ1* and *cepA* in CRKP isolates. Specific clonal dissemination of CRKP was detected within the same hospital or between different hospitals. Therefore, medical institutions should choose and use disinfectants correctly to prevent the spread of CRKP.

## INTRODUCTION

Carbapenem-resistant *Klebsiella pneumoniae* (CRKP) infection is rapidly emerging as a life-threatening nosocomial disease in many countries. In 2016, the WHO was asked to create a priority list of antibiotic-resistant bacteria to support research and development of effective drugs. As expected, CRKP was listed as one of the critical priority bacteria,^1^ whereas carbapenem-resistant Enterobacterales was listed as an urgent threat by the U.S. Centers for Disease Control and Prevention. According to the China Antimicrobial Surveillance Network, carbapenem resistance among *K. pneumoniae* increased significantly from 18.6% to 64.1% in 2018. From 2005 to 2018, the resistance rates of *K. pneumoniae* to imipenem and meropenem increased from 3.0% to 25% and 2.9% to 26.3%, respectively.^2^ Mortality rates for CRKP infections are higher than those for patients infected with carbapenem-susceptible *K. pneumoniae* (CSKP); pooled mortality was 42.1% among 2,462 patients infected with CRKP versus 21.2% in those infected with CSKP.^3^^,^^4^ Prudent use of antimicrobials, reflected also in the choice of antimicrobial combinations for treatment as well as efficient in-hospital control practices including meticulous hand hygiene, cleaning, and disinfection, are needed to control the spread of CRKP within hospitals.^5^^,^^6^

In clinical practice, decontamination and disinfection are the most important intervention measures to prevent bacterial spread. Disinfectants are widely acknowledged for removing microorganisms from the surface of objects and transmission media. Over the years, the excessive use of disinfectants has imposed selective pressure on strains, causing a wide distribution of disinfectant resistance genes.^7^ Many disinfectant resistance genes have been confirmed in multidrug-resistant bacteria, such as *qacA/B*, *qacE*, *qacEΔ1*, *qacG*, *qacJ*, *cepA*, *arcA,* and *kdeA*.^8^ In gram-negative bacteria, the plasmid-encoded *qacE*, *qacEΔ1*, *qacF,* and *qacG* genes of the SMR family are associated with quarternary ammonium compound protection. Among them, *qacEΔ1* seems to be widely disseminated.^9^ Another mechanism of biocide tolerance is related to the chromosomally encoded *cepA* exporter, which confers chlorhexidine protection in *K. pneumoniae*.^10^ However, the emergence of disinfectant resistance has become a severe threat to safety and health and to the rational allocation of resources. The genes *cepA* and *qacEΔ1* are closely associated with decreasing antiseptic susceptibility in strains of *K. pneumoniae*.^8^^,^^11^

There is still a lack of multicenter study on CRKP resistance to disinfectants in Wuhan, China. Therefore, the aim of this study was to identify the genetic characterization of these genes in CRKP and to investigate the diversity of the gene cassettes.

## MATERIALS AND METHODS

### Bacterial strains.

From June 2018 to March 2019, we collected a total of 74 nonduplicate clinical isolates of CRKP from six hospitals in Wuhan, the capital of Hubei province, China. The CRKP strains were defined as having a minimum inhibitory concentration (MIC) greater than 4 µg/mL for imipenem and meropenem or greater than 2 µg/mL for ertapenem. The isolates were obtained from various clinical specimens, including sputum, urine, blood, secretion, and drainage. Three of the hospitals were tertiary hospitals, including Wuhan No. 1 Hospital (31 isolates), Hubei Maternal and Child Health Care Hospital (18 isolates), and Wuhan Fourth Hospital (4 isolates). The remaining three hospitals were secondary hospitals, including Huangpi People’s Hospital (13 isolates), Wuhan Hankou Hospital (5 isolates), and the First People’s Hospital of Jiangxia District (3 isolates). Isolate identification and antimicrobial susceptibility testing were performed by laboratory personnel of the respective hospitals, and bioinformation of all strains was reviewed by using the Vitek 2 Compact System (bioMérieux, Lyons, France) in the laboratory of the Wuhan Center for Disease Control and Prevention. The CRKP strains were stored in brain heart infusion broth supplemented with 20% glycerol at −20°C for further analysis. *Escherichia coli* ATCC25922 and *Salmonella* H9812 were taken as quality control strains for susceptibility testing and pulsed-field gel electrophoresis (PFGE), respectively.

The study was approved by the Ethics Committee of the Wuhan Center for Disease Control and Prevention (WHCDCIRB-K-2021038).

### Antimicrobial susceptibility testing.

Antimicrobial susceptibilities for all isolates were initially detected by using gram-negative susceptibility cards on the Vitek 2 Compact System. We evaluated the susceptibility of 18 antibiotics, including ampicillin, amoxicillin/clavulanic acid, piperacillin, cefazolin, ceftazidime, ceftriaxone, cefepime, aztreonam, ertapenem, imipenem, meropenem, amikacin, gentamicin, ciprofloxacin, levofloxacin, tetracycline, nitrofurantoin, and trimethoprim/sulfamethoxazole. Susceptibility testing results were interpreted according to the criteria recommended by the Clinical and Laboratory Standards Institute.^12^

### Real-time polymerase chain reaction (PCR).

We designed primers for the well-known disinfectant resistance genes *qacEΔ1* and *cepA* by using Primer5.0 software (PREMIER Biosoft, Palo Alto, CA), according to the various gene sequences published in the GenBank database (https://blast.ncbi.nlm.nih.gov/Blast.cgi). All real-time PCR primers and probes targeting sequences used in this study are listed in Table 1. We extracted bacterial DNA from CRKP isolates by using a bacterial DNA extraction kit (Qiagen, Beijing, China). Real-time PCR (Roche, Lightcycler 480, Basel, Switzerland) conditions were set as follows: 95°C for 10 minutes, followed by 40 sequential cycles of 95°C for 10 seconds, 58°C for 30 seconds, and 72°C for 1 second. *QacEΔ1*-producing *K. pneumoniae* HP6 and *cepA-*carrying *K. pneumoniae* HK14 acted as positive controls. *QacEΔ1-*/*cepA*-negative isolate (*K. pneumoniae* FY35) and distilled water were negative and blank controls, respectively.

**Table 1:** Real-time PCR primers and probe for amplifying disinfectant resistance genes

Gene	Objects	Sequences and modifications (5′→3′)
*qacEΔ1*	Primer-F	CAGCCATTGCCTGGTTGC
	Primer-R	CGCAGCGACTTCCACGAT
	Probe	FAM-CCATACCTACAAAGCCCCACGCATC-BHQ1
*cepA*	Primer-F	GCGGGCGGATATGCTTCATT
	Primer-R	ATGCCAGCCGTACCAGGATA
	Probe	FAM-ATGATGAACGGCGCCATTCTGGTGGCG-BHQ1

F = forward; PCR = polymerase chain reaction; R = reverse.

### Pulsed-field gel electrophoresis.

The genotypes of CRKP isolates co-harboring *qacEΔ1* and *cepA* were determined by PFGE analysis. Pulsed-field gel electrophoresis analysis was performed as previously described using the XbaI restriction endonuclease (TAKARA, Shiga, Japan). The running parameters were set as follows: an initial pulse of 6 seconds, a final pulse of 36 seconds, at 6 V/cm for 18.5 hours at 14°C. The gels were analyzed using BioNumerics version 7.6 (Applied Maths, Sint-Martens-Latem, Belgium), and cluster analysis and phylogenetic trees were subsequently prepared. The similarity of the PFGE banding patterns was calculated using the Dice coefficient, and isolates with a PFGE profile exhibiting more than 80% similarity were considered closely related strains.^13^

### Data analysis.

Data analysis was conducted using IBM SPSS version 22.0 software (SPSS Inc., Chicago, IL) and BioNumerics version 7.6 (Applied Maths). Categorical variables were summarized by absolute frequencies and percentages and continuous variables by medians and ranges. The χ^2^ test and Fisher’s exact test were used to compare proportions, where appropriate. All tests were two-tailed, and *P* values less than 0.05 were considered statistically significant.

## RESULTS

### Bacterial isolates.

A total of 74 CRKP isolates were collected from 74 inpatients. The median age of the patients was 69.5 years, with the youngest patient being a 16-day-old baby and the eldest being a 93-year-old patient. Of the 74 isolates, 21 were collected from three secondary hospitals, and 53 were from three tertiary hospitals. Forty-nine isolates were from the intensive care unit (ICU), 15 isolates from the medical ward, three isolates from the surgical ward, and seven isolates from the pediatric ward. Most of the isolates were recovered from sputum (45.9%, 34/74), followed by urine (14.9%, 11/74), and blood (12.2%, 9/74).

### Antimicrobial susceptibility.

Table 2 shows the results of antimicrobial susceptibility. The rates of resistance to carbapenems were 93.2% for ertapenem, 90.5% for imipenem, and 87.8% for meropenem, with all isolates being resistant to at least one carbapenem antibiotic. The CRKP isolates exhibited high rates of resistance to the majority of antibiotics, with resistance rates exceeding 90%. The rate of susceptibility to tetracycline was only 52.7%.

**Table 2:** Antimicrobial susceptibility testing results of the 74 CRKP isolates

Antibiotics	Susceptibility testing (%)		
	Resistant	Intermediate	Susceptible
Ampicillin	74 (100)	0	0
Amoxicillin/clavulanic acid	71 (95.9)	1 (1.4)	2 (2.7)
Piperacillin	68 (91.9)	2 (2.7)	4 (5.4)
Cefazolin	72 (97.3)	0	2 (2.7)
Ceftazidime	70 (94.6)	0	4 (5.4)
Ceftriaxone	70 (94.6)	0	4 (5.4)
Cefepime	64 (86.5)	0	10 (13.5)
Aztreonam	68 (91.9)	0	6 (8.1)
Ertapenem	69 (93.2)	0	5 (6.8)
Imipenem	67 (90.5)	2 (2.7)	5 (6.8)
Meropenem	65 (87.8)	3 (4.1)	6 (8.1)
Amikacin	50 (67.6)	0	24 (32.4)
Gentamicin	58 (78.4)	0	16 (21.6)
Ciprofloxacin	60 (81.0)	3 (4.1)	11 (14.9)
Levofloxacin	60 (81.0)	3 (4.1)	11 (14.9)
Tetracycline	25 (33.8)	10 (13.5)	39 (52.7)
Nitrofurantoin	60 (81.1)	6 (8.1)	8 (10.8)
Trimethoprim/ sulfamethoxazole	43 (58.1)	0	31 (41.9)

CRKP = carbapenem-resistant *Klebsiella pneumoniae.*

### Molecular characteristics.

Table 3 presents the results of PCR analysis for all 74 CRKP isolates. Of these isolates, 48 (64.9%) were positive for *qacEΔ1* and 69 (93.2%) were positive for *cepA*. The prevalence of *cepA* was significantly higher than that of *qacEΔ1* (χ^2^ = 17.0, *P* < 0.05). *qacEΔ1* and *cepA* were co-detected in 46 isolates (62.2%), whereas only three isolates (4.1%) lacked both disinfectant resistance genes. The detection rate of *qacEΔ1* in tertiary hospitals (69.8%) was higher than that in secondary hospitals (52.4%). In terms of hospital wards, the detection rate of *qacEΔ1* was 67.3% in the ICU, 60.0% in the medical ward, and 85.7% in the pediatric ward, but it was not detected in the surgical ward (χ^2^ = 8.1, *P* < 0.05). The detection rate of *qacEΔ1* in different specimen types was similar, with no significant differences (χ^2^ = 7.8, *P* > 0.05).

**Table 3:** The detection rate of *qacEΔ1* and *cepA* in CRKP isolates

Types	No. of strains	Disinfectant resistance gene (%)		
		*qacEΔ1*	*cepA*	*qacEΔ1 + cepA*
Hospital level				
Secondary hospital	21	11 (52.4)	18 (85.7)	10 (47.6)
Tertiary hospital	53	37 (69.8)	51 (96.2)	36 (67.9)
Ward				
ICU*	49	33 (67.4)	47 (95.5)	32 (65.3)
Medical ward†	15	9 (60.0)	13 (86.7)	9 (60.0)
Surgical ward‡	3	0	3 (100)	0
Pediatric ward§	7	6 (85.7)	6 (85.7)	5 (71.4)
Specimen				
Sputum	34	24 (70.6)	32 (94.1)	22 (64.7)
Blood	9	5 (55.6)	9 (100)	5 (55.6)
Urine	11	6 (54.6)	10 (90.9)	6 (54.6)
Secretion	7	5 (71.4)	7 (100)	5 (71.4)
Drainage	5	1 (20.0)	3 (60.0)	1 (20.0)
Other location‖	8	7 (87.5)	8 (100)	7 (87.5)

CRKP = carbapenem-resistant *Klebsiella pneumoniae.*

* Included intensive care unit (ICU), neurology ICU, and pediatric ICU.

† Included respiratory medicine, oncology, recovery unit, traditional Chinese medicine, nephrology, infectious diseases department, neurology, and cardiology.

‡ Included gastrointestinal surgery and neurosurgery.

§ Included pediatric cardiology, neonatology, and endocrine genetics in children.

‖ Included bronchoalveolar lavage fluid and bronchoscopy.

Table 4 presents the results of antimicrobial resistance rates between disinfectant resistance gene–positive and –negative strains. The genes of *cepA* and *qacEΔ1* are closely associated with increasing antimicrobial resistance in part of the CRKP strains. The *qacEΔ1*-positive strains showed higher resistance rates to piperacillin, amoxicillin/clavulanic acid, aztreonam, amikacin, gentamicin, ciprofloxacin, levofloxacin, and nitrofurantoin (all *P* < 0.05). The *cepA*-positive strains showed higher resistance rates to piperacillin, ceftazidime, ceftriaxone, ertapenem, imipenem, ciprofloxacin, and levofloxacin (all *P* < 0.05).

**Table 4:** Antimicrobial resistance rates between disinfectant resistance gene–positive and –negative strains

Antibiotics	Resistant (%)				*P* *	*P* †
	*qacEΔ1-*positive	*qacEΔ1*-negative	*CepA-*positive	*CepA*-negative		
	(*N = *48)	(*N *= 26)	(*N *= 69)	(*N = *5)		
Ampicillin	48 (100)	26 (100)	69 (100)	5 (100)	1.00‡	1.00‡
Amoxicillin/clavulanic acid	48 (100)	23 (88.5)	66 (95.7)	5 (100)	< 0.05‡	1.00‡
Piperacillin	47 (97.9)	21 (80.8)	66 (95.7)	2 (40.0)	< 0.05‡	< 0.01‡
Cefazolin	48 (100)	24 (92.3)	67 (97.1)	5 (100.0)	0.12‡	1.00‡
Ceftazidime	47 (97.9)	23 (88.5)	67 (97.1)	3 (60.0)	0.24§	< 0.05‡
Ceftriaxone	47 (97.9)	23 (88.5)	67 (97.1)	3 (60.0)	0.24§	< 0.05‡
Cefepime	41 (85.4)	23 (88.5)	61 (88.4)	3 (60.0)	0.99§	0.13‡
Aztreonam	47 (97.9)	21 (80.8)	65 (94.2)	3 (60.0)	< 0.05§	0.05‡
Ertapenem	47 (97.9)	22 (84.6)	66 (95.7)	3 (60.0)	0.09§	< 0.05‡
Imipenem	46 (95.8)	21 (80.8)	65 (94.2)	2 (40.0)	0.06‡	< 0.01‡
Meropenem	44 (91.7)	21 (80.8)	62 (89.9)	3 (60.0)	0.21‡	0.06‡
Amikacin	41 (85.4)	9 (34.6)	48 (69.6)	2 (40.0)	< 0.01‖	0.39§
Gentamicin	45 (93.8)	13 (50.0)	56 (81.2)	2 (40.0)	< 0.01‖	0.11§
Ciprofloxacin	45 (93.8)	15 (57.7)	59 (85.5)	1 (20.0)	< 0.01‡	< 0.01‡
Levofloxacin	42 (87.5)	15 (57.7)	57 (82.6)	0	< 0.01‡	< 0.01‡
Tetracycline	15 (31.3)	10 (38.5)	23 (33.3)	2 (40.0)	0.79‡	0.69‡
Nitrofurantoin	43 (89.6)	17 (65.4)	57 (82.6)	3 (60.0)	< 0.05‡	0.24‡
Trimethoprim/sulfamethoxazole	31 (64.6)	12 (46.2)	39 (56.5)	4 (80.0)	0.13‖	0.58§

* Statistical analysis between *qacEΔ1*-positive and -negative.

† Statistical analysis between *CepA*-positive and -negative.

‡ Fisher’s exact test.

§ Continuity correction.

‖ Likelihood ratio.

### Pulsed-field gel electrophoresis typing.

**Figure 1.**
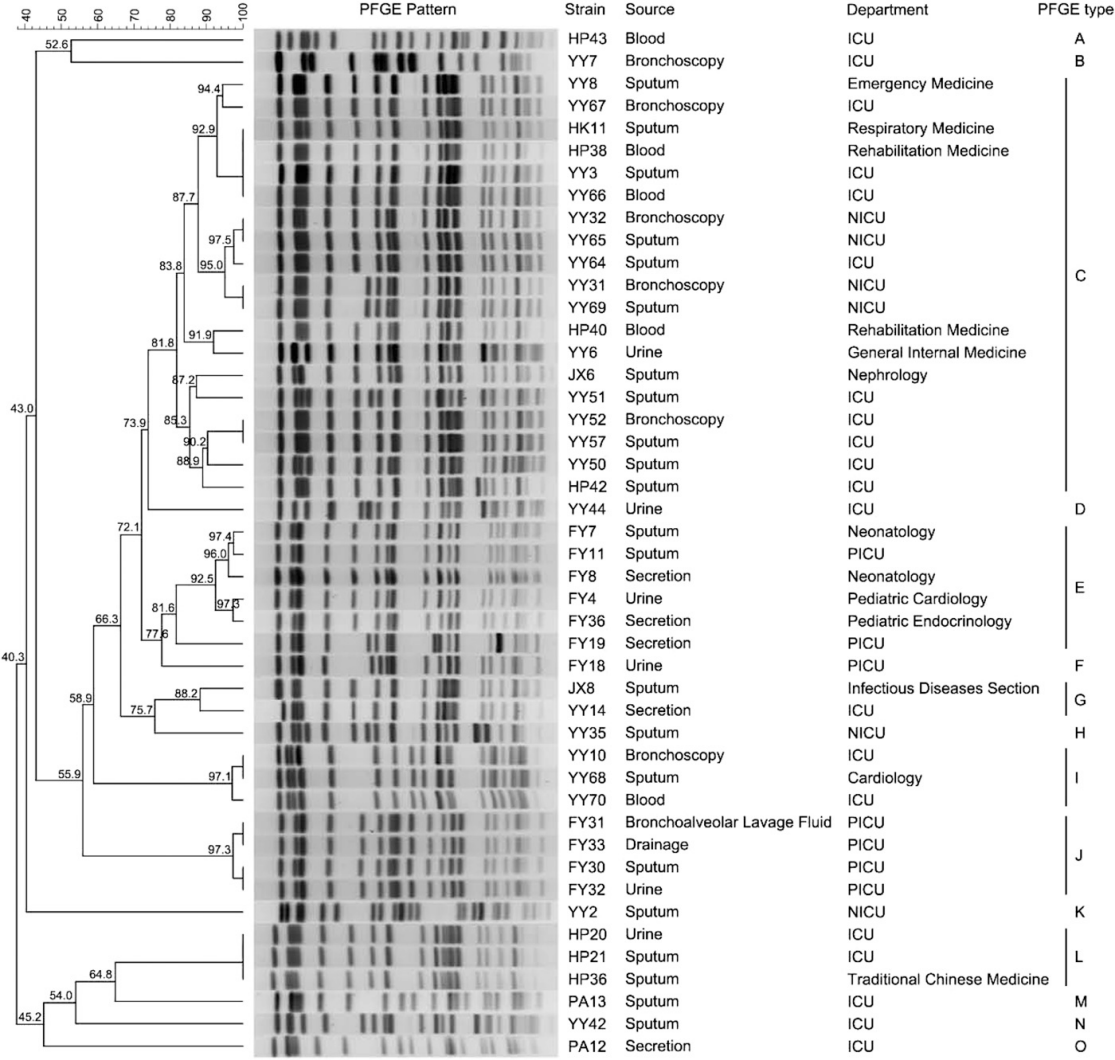
A dendrogram of PFGE pattern for 46 CRKP strains. These isolates were divided into 15 different clonal clusters (Types A to O) using 80% similarity as the cutoff. CRKP = carbapenem-resistant *Klebsiella pneumoniae*; ICU = intensive care unit; NICU = neurology intensive care unit; PFGE = pulsed-field gel electrophoresis; PICU = pediatric intensive care unit.

The dendrogram generated from the PFGE image revealed that the 46 CRKP strains co-producing *qacEΔ1* and *cepA* were divided into 15 different clonal clusters (designated as Types A to O) using 80% similarity as the cutoff (Figure 1). The most common clonal clusters were Type C (41.3%), Type E (13.0%), Type J (8.7%), Type I (6.5%), and Type L (6.5%). Type C, the predominant cluster, consisted of 19 strains from patients in four different hospitals. Among these, YY52 and YY57, YY31 and YY69, YY32 and YY65, HK11, HP38, YY3, and YY66 showed identical patterns (100%), suggesting that they were the same strain, whereas other isolates displayed similar but not identical band patterns (80%), indicating that they may belong to the same clonal group. Type E, the second-most common cluster, included six isolates from patients in three different departments of the same hospital. Among the Type J isolates, four collected from the pediatric intensive care unit (PICU) of the same hospital, FY31, FY33, FY30 and FY32 were all identical patterns (100%); further analysis found that four patients were hospitalized from November 5–22, 2018. The Type L isolates, HP20, HP21, and HP36, collected from the ICU and traditional Chinese medicine departments of the same hospital, were found to be the same strain. However, nine CRKP isolates exhibited unrelated PFGE patterns, suggesting that the dissemination of *qacEΔ1* and *cepA* genes occurred horizontally throughout the population rather than by the spread of a single strain.

## DISCUSSION

Owing to variations in natural environments and economic levels across different regions, the implementation of disinfection measures and the type of antibiotics used in hospitals differ, resulting in varied disinfectant resistance mechanisms among strains from different regions. Therefore, it is important to base hospital infection control measures on local epidemiological studies. For this study, we selected six different hospitals in Wuhan, including both secondary and tertiary hospitals and both general and specialized hospitals. To the best of our knowledge, this is the first multicenter study to report on disinfectant-resistance genes and genetic relationships of CRKP isolates from inpatients in Wuhan, China.

Disinfectants are widely acknowledged for removing microorganisms from the surface of objects and transmission media. However, the emergence of disinfectant resistance has become a severe threat to life and health and the rational allocation of resources because of reduced disinfectant effectiveness. Bacteria can develop disinfectant resistance through the expression of disinfectant-resistant genes.^14^ The presence of the *qacEΔ1* and *cepA* genes is known to be associated with high-level MICs of biocides.^8^ Previous studies have reported that the *qacEΔ1* and *cepA* genes have a close relationship with decreasing disinfectant susceptibility in *K. pneumoniae* strains.^15^ However, it is important to note that the *qacEΔ1* gene is a defective gene, and various mechanisms are involved in biocide resistance.^16^ In our study, we investigated the presence of the two disinfectant resistance genes *qacEΔ1* and *cepA* in CRKP isolates.

Our findings revealed that 62.2% of the CRKP strains co-harbored *qacEΔ1* and *cepA*. Specifically, among the clinical isolates of CRKP, 64.9% and 93.2% were positive for *qacEΔ1* and *cepA*, respectively. The *cepA* gene was much more prevalent than the *qacEΔ1* gene. These results are consistent with previous studies. Abuzaid et al. reported that the *cepA* gene was carried by 56 (87.5%) *K. pneumoniae* isolates,^7^ and Chen et al. reported that 41.7% were positive for *qacEΔ1* and 80.6% for *cepA* among 36 CRKP strains at a tertiary hospital in China.^16^

In contrast to our results, a previous study conducted in Iran reported lower prevalence rates of the *qacEΔ1* and *cepA* genes among clinical isolates of *K. pneumoniae*, with the *qacEΔ1* gene detected in 30.6% of the clinical isolates and the *cepA* gene found in 22.4%.^17^ Our results indicate that the *qacEΔ1* and *cepA* genes are widely distributed among CRKP isolated from hospitals, with an increase in their prevalence among CRKP strains in China.

The antimicrobial susceptibility profile showed that all CRKP strains were multidrug-resistant, as they were nonsusceptible to at least one antibiotic from three or more antibiotic classes. All 74 strains exhibited high rates of resistance to the majority of antibiotics, which indicates that the drug resistance situation of CRKP strains in Wuhan is very severe, which is consistent with some previous studies.^18^^–^^20^ The results showed that the *qacEΔ1* and *cepA* genes are closely associated with increasing antimicrobial resistance in part of the CRKP strains, such as piperacillin, ciprofloxacin, and levofloxacin. The *qacEΔ1* gene appears to be part of a small resistance island, suggesting that this gene is linked to and migrated with antibiotic resistance genes.^13^ The widespread carriage of *qacEΔ1* genes in CRKP and their linkage to antibiotic resistance suggests that widespread use of disinfectants could select antibiotic-resistant strains, though there is no direct evidence for this so far.^7^^,^^13^ The relationship between the existence and expression of disinfectant genes and their resistance to bacteria needs further study. Pulsed-field gel electrophoresis is considered the “gold standard” for bacterial typing.^21^ Recently, matrix-assisted laser desorption/ionization time of flight mass spectrometry and whole-genome sequencing have also been proposed for bacterial typing.^22^ Whole-genome sequencing, in particular, provides more information and has been proposed as an alternative method for bacterial typing. However, PFGE remains an affordable and relevant technique in small laboratories and hospitals, especially in developing countries. With its high discriminatory power, PFGE is useful in outbreak investigations, surveillance, and infection control.^23^ In this study, the PFGE data revealed that the 46 CRKP isolates were divided into 15 distinct clonal clusters. The major cluster Type C included 19 strains from patients in four different hospitals, indicating a possible epidemiological link between the hospitals, potentially due to patient or staff transfer. Similarly, the second major cluster, Type E, included six isolates from patients in three different departments within the same hospital, indicating that CRKP can spread within the same hospital between different departments. Type J isolates collected from the same hospital’s PICU revealed that CRKP can also spread among different patients in the same department. These findings highlight the considerable DNA polymorphism of CRKP and suggest that polyclonal dissemination is a significant factor in the spread of CRKP.

In conclusion, the appropriate use of biocides is essential for the effective prevention and control of CRKP infections. However, our study showed a high frequency of *qacEΔ1* and *cepA* in CRKP isolates, indicating the potential for biocide resistance and a higher level of antibiotic resistance in CRKP. Therefore, monitoring the disinfectant resistance rate of CRKP strains in the hospital environment should be a priority. This will ensure that appropriate and effective disinfection measures are implemented to prevent the spread of these life-threatening resistant strains.
